# AQP1 suppression by ATF4 triggers trabecular meshwork tissue remodelling in ET‐1‐induced POAG

**DOI:** 10.1111/jcmm.15032

**Published:** 2020-02-13

**Authors:** Yingying Zhao, Huazhang Zhu, Yangfan Yang, Yiming Ye, Youli Yao, Xiaoyan Huang, Yixiang Zhang, Xingsheng Shu, Xianxiong Chen, Yatao Yang, Junxian Ma, Le Cheng, Xiaomei Wang, Ying Ying

**Affiliations:** ^1^ Department of Physiology School of Basic Medical Sciences School of Medicine Shenzhen University Shenzhen China; ^2^ State Key Laboratory of Ophthalmology Zhongshan Ophthalmic Center Sun Yat‐Sen University Guangzhou China; ^3^ Department of Urology Shenzhen People's Hospital The Second Affiliated Hospital of Jinan University Shenzhen China; ^4^ School of information engineering Shenzhen University Shenzhen China; ^5^ BGI‐Yunnan BGI‐Shenzhen Kunming China

**Keywords:** AQP1, ATF4, POAG, suppression, tissue remodelling, trabecular meshwork, transcriptional

## Abstract

Primary open‐angle glaucoma (POAG) is the second leading cause of irreversible blindness worldwide. Increased endothelin‐1 (ET‐1) has been observed in aqueous humour (AH) of POAG patients, resulting in an increase in the out‐flow resistance of the AH. However, the underlining mechanisms remain elusive. Using established in vivo and in vitro POAG models, we demonstrated that water channel Aquaporin 1 (AQP1) is down‐regulated in trabecular meshwork (TM) cells upon ET‐1 exposure, which causes a series of glaucomatous changes, including actin fibre reorganization, collagen production, extracellular matrix deposition and contractility alteration of TM cells. Ectopic expression of AQP1 can reverse ET‐1‐induced TM tissue remodelling, which requires the presence of β‐catenin. More importantly, we found that ET‐1‐induced AQP1 suppression is mediated by ATF4, a transcription factor of the unfolded protein response, which binds to the promoter of *AQP1* and negatively regulates *AQP1* transcription. Thus, we discovered a novel function of ATF4 in controlling the process of TM remodelling in ET‐1‐induced POAG through transcription suppression of AQP1. Our findings also detail a novel pathological mechanism and a potential therapeutic target for POAG.

## 1 INTRODUCTION

Primary open‐angle glaucoma (POAG) is the most common form of glaucoma and the second leading cause of blindness worldwide.^1^ Progressive loss of ganglion cell axons is attributed mostly to elevated intraocular pressure (IOP). The conventional AH drainage system is composed of a porous structured trabecular meshwork (TM) and Schlemm's canal. Resistance to aqueous out‐flow directly affects IOP. High drainage resistance is commonly associated with POAG. Upon exposure to mechanical strain and biochemical signals, a series of intracellular and extracellular morphogenetic changes affect pore formation and flexibility of TM tissue, including cytoskeleton rearrangement, collagen production and extracellular matrix (ECM) deposition. Chronic pathological changes result in an increase of irreversible drainage resistance and elevated IOP.

Endothelin‐1 (ET1) is a small peptide that is produced by the vascular endothelium, which regulates both the contractility^2^, ^3^ and permeability of the endothelium.^4^, ^5^ As a potent vasoconstrictor, elevated ET‐1 has been mainly implicated in many cardiovascular diseases. In the eye, ET‐1 can be synthesized and secreted by the vascular endothelium, retinal pigmented epithelium and non‐pigmented ciliary epithelium.^6^, ^7^, ^8^ Intriguingly, elevated ET‐1 has been observed in the AH of glaucoma eyes^9^ and significant correlation between IOP and ET‐1 concentration in AH of POAG patients has been found.^10^, ^11^ Accumulating evidence has revealed that ET‐1 plays an important role in the induction of TM contraction^12^, ^13^ and dysregulation of AH out‐flow dynamics, which results in IOP elevation.^14^, ^15^, ^16^, ^17^, ^18^, ^19^ It is evident that ET‐1, as a vasoconstrictor, can function in the endothelia of TM tissue and cause IOP elevation. However, the detailed molecular mechanism remains unclear.

Water channels are a class of plasma membrane proteins that mainly have functions in epithelial fluid transportation and intracellular water homeostasis. In the human eye, AQP1 is expressed in the iris and ciliary epithelium and mainly functions in AH production by transporting water out of the ciliary epithelium. Mice lacking AQP1 showed reduced aqueous fluid production and reduction of IOP.^20^ AQP1 is also expressed in trabecular meshwork cells and has been shown to regulate cell volume of the trabecular meshwork cells,^21^, ^22^ as well as other types of cells.^23^, ^24^, ^25^ However, the role, if any, of AQP1 in regulating the physiology of TM tissue in the pathogenesis of POAG remains unclear.

In this study, using in vivo and in vitro models, for the first time we demonstrated that water channel Aquaporin 1 (AQP1) expression is down‐regulated in trabecular meshwork cells, upon ET1 exposure, and that this contributes to a series of glaucomatous changes including actin fibre reorganization, collagen production, ECM deposition and contractility alteration of TM cells. This TM tissue remodelling process is mediated by the interaction between AQP1 and β‐catenin. More importantly, we found that ET‐1‐induced suppression of AQP1 is mediated by ATF4, a transcription factor of the unfolded protein response that binds to the promoter of the *AQP1* gene and negatively regulates its transcription. We also address the hypothesis that during the early stages of glaucoma, increased humous levels of ET‐1 mediates trabecular meshwork tissue remodelling, and we discovered a novel function of ATF4 in controlling the process of TM remodelling through transcription suppression of AQP1 in POAG.

## 2 MATERIALS AND METHODS

### 2.1 Animals and cells

Male NZW white rabbits aged 12 weeks were purchased from Guangdong Medical Laboratory Animal Center. Experimental animals were housed in individual cages and topical ocular ET‐1 (2 μmol/L) or PBS eye drops were applied 3 times a day for 2 weeks and IOPs were recorded using opthalmotonometer weekly. Animals were killed at end of 2 weeks, and TM tissue were collected and fixed in 4% paraformaldehyde. Animal maintenance and experiment procedure were approved by the Laboratory Animal Ethics Committee of Shenzhen University.

The primary human TM cell line was kindly provided by Dr Minbin Yu^26^ and was confirmed by testing the mRNA level of CHI3L1 (Figure S1). Primary human TM cells were grown in Fibroblast Medium (Catalogue No. 2301; ScienCell Research Labs) and were used at the third to sixth passage. The cells were incubated at 37°C in a 5% CO2 environment. To identify the primary TM cells, the expression of known markers, Matrix Gla Protein, Chitinase‐3‐Like‐1, dexamethasone‐induced cross‐linked actin networks (CLANs) and up‐regulation of myocilin were analysed using immunofluorescence staining; the mRNA levels of Matrix Gla Protein, Chitinase‐3‐Like‐1 and dexamethasone‐induced up‐regulation of myocilin were also determined using RT‐PCR.

### 2.2 Immunohistochemistry

TM tissues were immunostained using antibodies indicated in Table 1. Images were obtained with an LSM 510 (Zeiss, Oberkochen, Germany) confocal microscope.

**Table 1:** Primer pairs and the sequence

Primer name	Sequence(5′–3′)
AQP1‐1‐F	CCTTCCTCCCTTTGTGCTCC
AQP1‐1‐R	GAGTGGGGCCAGCTTGGAAG
AQP1‐2‐F	CCAAAGCCTATTAGAGCAAC
AQP1‐2‐R	GAGCCCTGGGCCCGGGGACT
AQP1‐3‐F	GGCCAGTGCCTCTCTGGGTC
AQP1‐3‐R	CTTCACCAGCCTGCCCTCAA
AQP1‐4‐F	ATTGGTTCAAATCGCTGTC
AQP1‐4‐R	GGGATCTGTCTCTTTCACTT
AQP1‐5‐F	CTTTTGCTCTATTTCCCTGC
AQP1‐5‐R	CCCTCCCGCTCTCTGCCTCTC
AQP1‐6‐F	TCATTTATCTCTCCCTTTCT
AQP1‐6‐R	CTCCCTTCTGGGCCTGGTTC
AQP1‐7‐F	TTCCAGCACGTTCCATGCA
AQP1‐7‐R	CTCTGCAGGATAGCCCCTGG
AQP1‐8‐F	AAGAGGCATAAGACCCACT
AQP1‐8‐R	GCTAAGAAAGTACTTATG
AQP1‐9‐F	CCTTCGGCCACCTGGGCT
AQP1‐9‐R	GAGAGAGCTCTGGTTGGACG
AQP1‐10‐F	GAAACAGTTTGCCCTCC
AQP1‐10‐R	AAACTGACCAGAGAGCTCCT
AQP1‐11‐F	GGCCTTCCTCCCTTTGTG
AQP1‐11‐R	CGTGAAGGTGTCGCTGGCC
AQP1‐12‐F	AACAACCAGACGGCGGTCCA
AQP1‐12‐R	ACGTGAGTGGGGTGTCCCT
AQP1‐13‐F	GGGAACTCGCTTGGCCGCA
AQP1‐13‐R	CTATTATGGGGAATAAGCT
AQP1‐14‐F	GCCAATGTCCCCTGCAGGGC
AQP1‐14‐R	CCCTGAGATTAGACAAA
AQP1‐15‐F	AAAGGCCCTCTTCCACTCT
AQP1‐15‐R	CCTGGCTTGAATGGGGTCT
AQP1‐16‐F	TTTGCAACACGTGCCCA
AQP1‐16‐R	AGGCCTGGGGGTGCCCTT
AQP1‐17‐F	GGCCCAAGGGACAGGTGAG
AQP1‐17‐R	TTAGCACTTCTGTGTCACGG
AQP1‐18‐F	GCTGGCTCAGCCTGGCCATG
AQP1‐18‐R	CGTCCCTGTAGGGGAGTGGAT
AQP1‐19‐F	TGGCTGGGTATGATGGGCTG
AQP1‐19‐R	ATGGGAGAGAAAGAGGAG

### 2.3 Immunofluorescence

The HTMCs were seeded on coverslips in a 24‐well plate with a density of 8X104 cells/well. After corresponding treatment, HTMCs were washed with pre‐warmed PBS and fixed with 4% paraformaldehyde. The cells were permeabilized with 0.1% Triton X‐100 (Sigma‐Aldrich) and blocked with 5% goat serum (Life Technologies). For staining for collagen I, cells were incubated overnight with primary antibodies, including anti‐collagen I (Abcam), and the cells were washed and incubated with Alexa 488‐conjugated secondary antibodies (Thermo Fisher Scientific). The cells were probed with Alexa Fluor 594 phalloidin (Thermo Fisher Scientific). Afterwards, the coverslips were mounted with DAPI (Thermo Fisher Scientific). The pictures were taken by the LSM 510 (Zeiss) confocal microscope.

### 2.4 ELISA

The supernatants were collected and collagen I and collagen III contents in the supernatants were measured using kits (R&D systems).

### 2.5 Collagen Gel Contraction Assays (CGC Assays)

Collagen gel contraction assays were performed with a Cell Contraction Assay Kit (CBA‐201, Cell Biolabs) according to the manufacturer's instructions, with minor modifications by Dr Minbin Yu.^26^

### 2.6 RNA isolation and Quantitative RT‐PCR(qPCR)

Total RNA was extracted using Trizol according to the manufacturer's instructions. The primers for target genes were obtained from the PrimerBank Database. QRT‐PCR was performed with the FastStart Universal SYBR Green Master reagent (Roche) and a Roche 480 real‐time PCR system. Target gene expression was calculated using the 2 (DDC[t]) method using b‐actin as the housekeeping gene. The results were presented as a relative value compared to the control group.

The primer sequences of AQP1 are as follows:

5′‐TGCCATCGGCCTCTCTGTA‐3′ (forward primer)
5′‐CAGGGTTAATCCCACAGCCA‐3′ (reverse primer)

### 2.7 Western blot

Treated and untreated HTMCs were rinsed with PBS and lysed in lysis buffer (Sigma) with 1X cocktail inhibitor (MERK USA). Cellular protein resolved by SDS‐PAGE was immunoblotted as previous study (Ying et al, 2012). Primary antibody for tested protein was listed as follows:

**Table:** 

Name	Host	Company	ID
AQP1	Rabbit	Millipore	AB2219
ATF4	Rabbit	Abcam	ab184909
COL1A1(Collagen I)	Rabbit	Boster Biological Technology	BA0325
COL3A1(Collagen III)	Mouse	Boster Biological Technology	BM1625
F‐actin	Mouse	Abcam	ab205
β‐catenin	Rabbit	Abcam	ab32572
elf2‐α	Rabbit	Cell singal technology	9722
Phospho‐eIF2α (Ser51)	Rabbit	Cell singal technology	3398
GAPDH	Rabbit	Cell singal technology	5174
Flag	Mouse	Abcam	ab18230

Immunoreactive bands were revealed by ECL and visualized by the KODAK Image Station 4000MM PRO.

### 2.8 RNA interference

To knock‐down of AQP1 in HTMCs, the following siRNA sequences (sense strands) and scrambled control were purchased from Dharmacon (ON‐TARGET plus Smart‐pool, Thermo Scientific):

1. 5′‐CCACGACCCTCTTTGTCTT ‐3′;
2. 5′‐GGAGGAGTATGACCTGGAT ‐3′;
3. 5′‐TTCTCCGAACGTGTCACGT ‐3′;

β‐catenin was knocked down with shRNA used in a previous study.^27^

To knock‐down of ATF4 in HTMCs, siRNA sense strands and scrambled negative control were purchased from Santa Cruz Biotechnology, CREB‐2 siRNA(h):35 112).

### 2.9 Protein Overexpression

Recombinant adenovirus coding AQP1 (Ad‐Flag‐AQP1) or for comparative control (Ad‐Flag) was constructed to express ZsGreen protein as a marker for the identification of infected cells.

### 2.10 Luciferase assay

The pRL‐TK vector containing Renilla luciferase gene was cotransfected as an internal reference to correct the transfection efficiency. Luciferase activity of negative control pGL3‐Basic vector was used. The significance of luciferase activity was measured using a Dual‐Luciferase Reporter System (Promega). The activity differences were analysed using one‐way ANOVA test. The values were averaged from three independent replicates. Error bars represent SD (n = 3). Diverse letters aside by the column are defined as statistically significant difference (*P* < .05).

### 2.11 Statistical analysis

Data analyses were used Graph Pad (GraphPad Prism). All data were expressed as mean ± SEM. Statistical significance was analysed by one‐way ANOVA followed by a Student's *t* test. Data were considered significant when *P* < .05.

## 3 RESULTS

### 3.1 ET‐1 induced pathological changes of POAG

**Figure 1:**
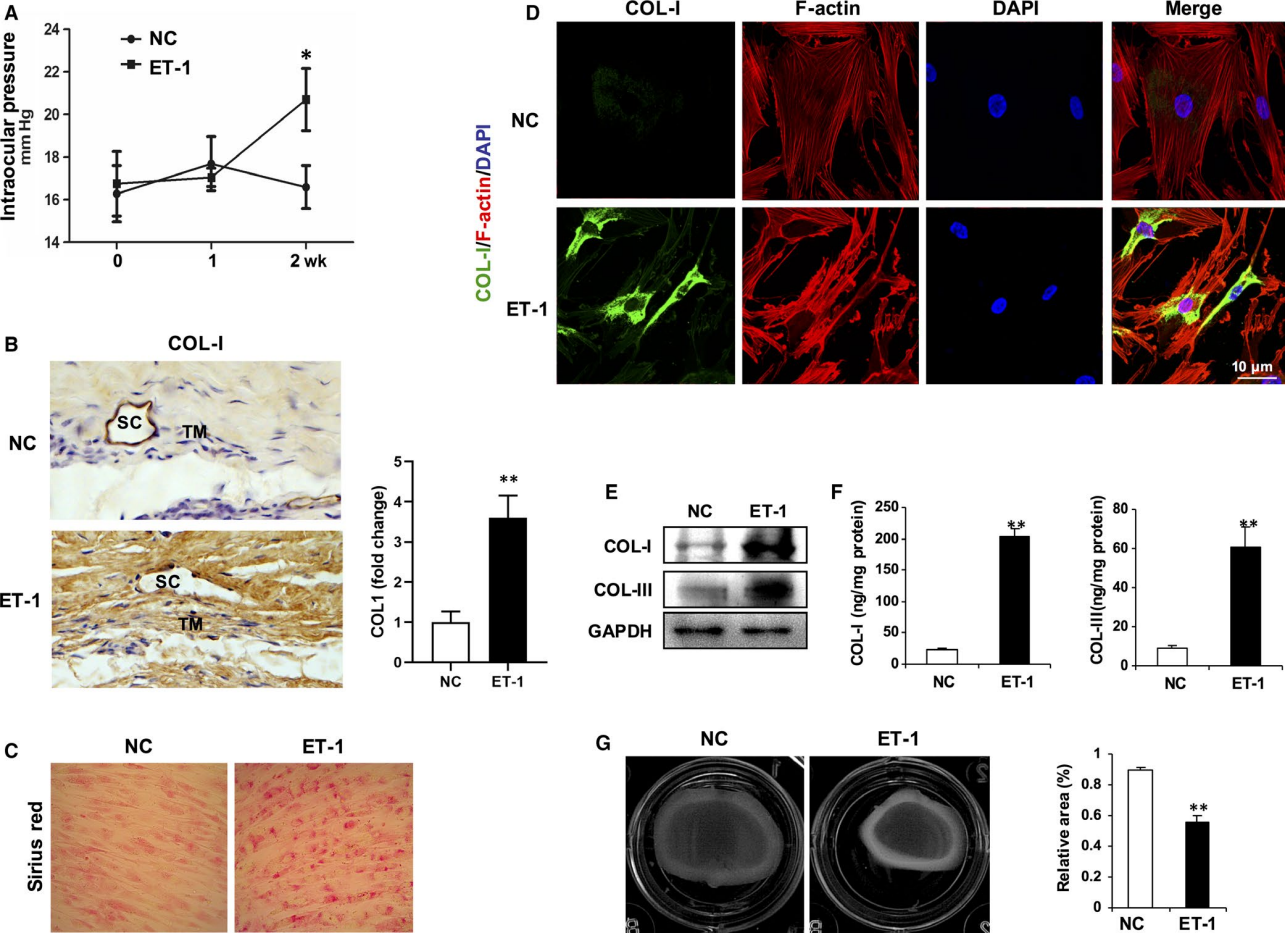
ET‐1 exposure mimics pathologic features of POAG. A, Elevated intraocular pressure (IOP) in ET‐1‐treated rabbit eyes. Topical application of ET‐1 (2 μmol/L) or PBS eye drops onto rabbit eyes 3 times a day for 2 wk, and IOPs were recorded weekly. Data are means ± SEM. n = 10 eyes per group, *P < .05 vs eyes applied with PBS (NC). B, Representative immunohistochemistry for collagen I (COL‐I) in sections of rabbit trabecular meshwork tissues. TM, trabecular meshwork; SC, Schlemm's canal. C, Deposition of collagen in cultured human trabecular meshwork cells (HTMCs) treated with ET‐1 (100 nmol/L) or PBS for 24 h were determined by Sirius Red staining. D, Representative immunofluorescence for collagen I (COL‐I) (green) or F‐actin (red). Nuclei were counterstained with DAPI (blue). Scale bar, 10 μm. E, Deposition collagen in cultured HTMCs were determined by Western blotting. F, Secretion of collagen in cultured HTMCs were determined by ELISA. Data are means ± SEM of three independent experiments. **P < .01 vs PBS control (NC). G, The contractility of HTMCs was determined by a collagen gel contraction assay. Representative images are shown. The area of the collagen disc was measured using Image J. The contraction degree was expressed as a percentage of the 24‐well area. The cultured HTMCs are from one human donor. Data are means ± SEM of three independent experiments. **P < .01 vs control

Given that ET‐1 contributes to the pathogenesis of POAG, we adopted an ET‐1 delivery strategy to establish models of POAG in vivo and in vitro. Topical administration of ET‐1 to rabbit eyes for 2 weeks led to significant IOP elevation (Figure 1A) and collagen deposition (Figure 1B), which represent the major pathological features of POAG. In order to further verify that ET‐1 can lead to cellular conformational change and collagen deposition, primary human TM cells from one human donor^26^ were treated with ET‐1 for 24 hours. As shown in Figure 1C, Sirius Red staining indicates heavy collagen deposition. Furthermore, both intracellular collagen production and extracellular collagen secretion of collagen I (COL‐I) and III (COL‐III) increased significantly in ET‐1‐treated HTMCs (Figure 1D‐F). The contractility features of TM cells directly affect AH drainage and IOP.^26^ We then measured the contractility of HTMCs using a gel contraction assay. A significant reduction in the area of collagen gels was observed in the ET‐1‐treated groups, compared with that of the untreated controls (Figure 1G), which is consistent with the results of previous studies.^17^, ^28^, ^29^ Thus, our data suggest that ET‐1 induced IOP elevation and collagen deposition mimics the pathological features of POAG. We then sought to investigate the potential molecular mechanisms underlying ET‐1‐induced POAG.

### 3.2 AQP1 is attenuated in ET‐1‐induced POAG models

**Figure 2:**
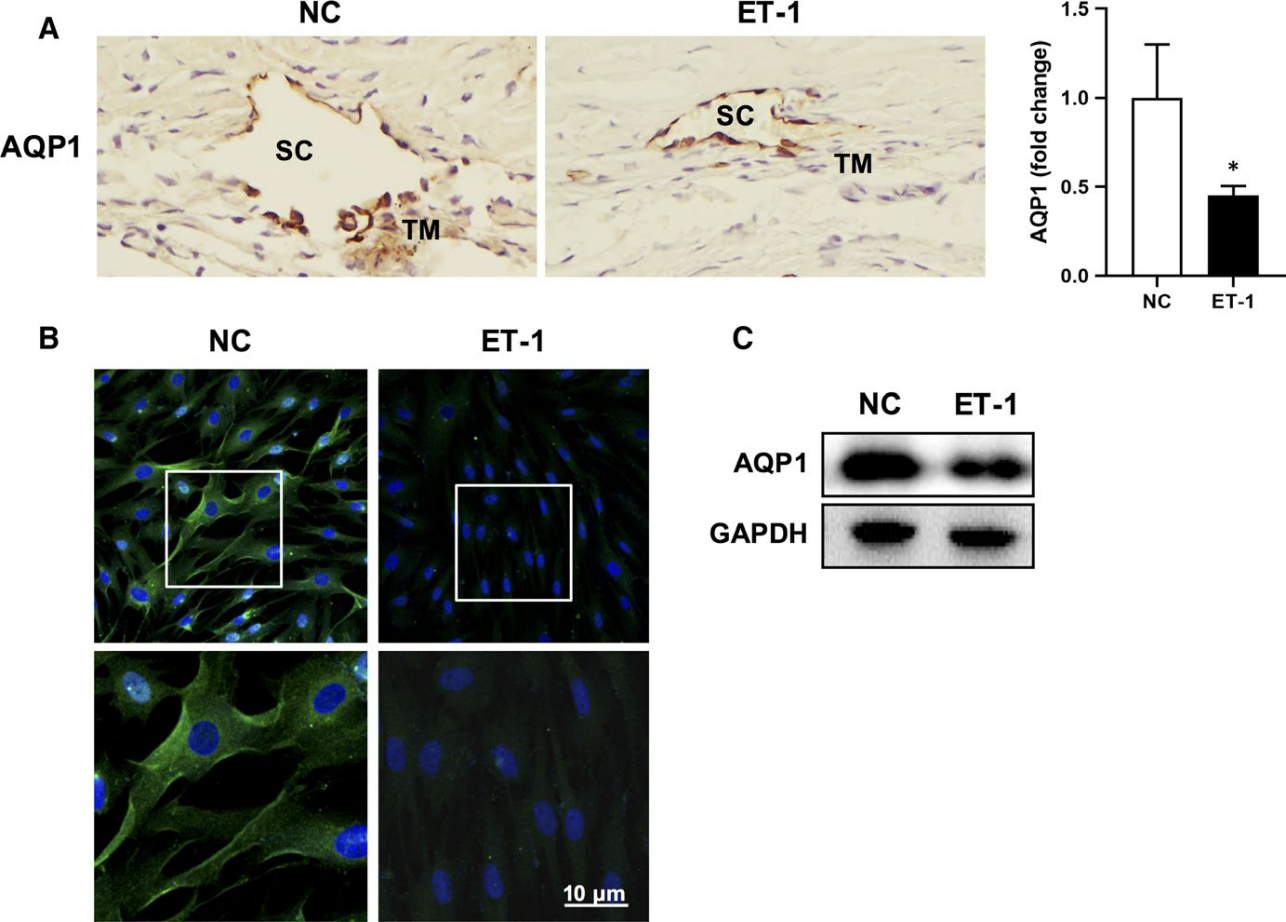
AQP1 level is attenuated in ET‐1‐induced POAG models. A, Representative immunohistochemistry for AQP1 in sections of rabbit trabecular meshwork tissues. TM, trabecular meshwork; SC, Schlemm's canal. B, Representative immunofluorescence staining for AQP1 (green, AQP1; blue, DAPI; scale bar, 10 μm) in HTMCs treated with ET‐1 or PBS, respectively. Bottom panels are high‐power magnification of the areas indicated by the boxes. C, Protein of AQP1 in HTMCs exposed to PBS or ET‐1 (100 nmol/L) for 24 h was determined by Western blot analysis. The cultured HTMCs are from one human donor

Initial in vivo ocular studies on rabbits showed that compared with age‐matched controls, water channel Aquaporin 1 (AQP1) was significantly attenuated in TM tissue of rabbits challenged with topical ocular ET‐1 (Figure 2A). Immunofluorescence staining consistently revealed a considerable decrease in AQP1 in ET‐1‐treated HTMCs in vitro (Figure 2B,C and Figure S2). These results suggest that AQP1 is attenuated in the TM of POAG induced by aqueous humour ET‐1.

### 3.3 AQP1 inhibits ET‐1‐induced F‐actin reorganization, elevated contractility and collagen deposition in HTMCs

**Figure 3:**
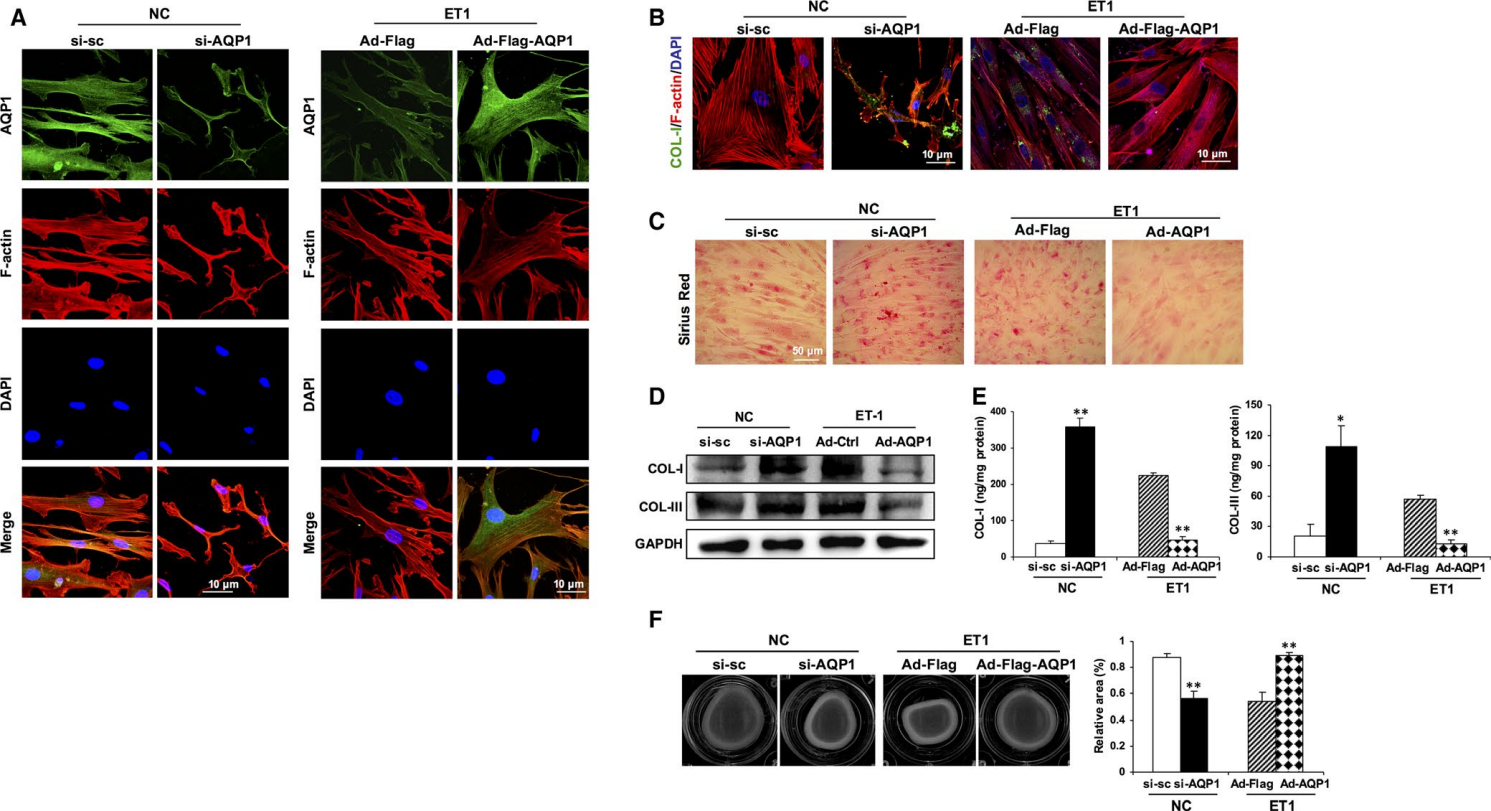
AQP1 inhibits ET‐1‐induced F‐actin reorganization, elevated contractility and collagen deposition in HTMCs. HTMCs were transfected with si‐AQP1 or scramble control si‐sc for 48 h in control HTMCs. Alternatively, cells were infected with an adenoviral vector expressing AQP1 (Ad‐Flag‐AQP1) or empty vector (Ad‐Flag). Twenty‐four hours later, cells were treated with ET‐1 for another 24 h. A, Representative immunofluorescence for AQP1 (green) and F‐actin (red). Nuclei were counterstained with DAPI (blue). Scale bar, 10 μm. B, Representative immunofluorescence for COL‐I (green) and F‐actin (red). Nuclei were counterstained with DAPI (blue). Scale bar, 10 μm. C, Representative images of Sirius Red staining. D, E, Protein expression or secretion of collagen I (COL‐I) and III (COL‐III) was determined by Western blot or ELISA, respectively. F, The contractility of HTMCs was determined by a collagen gel contraction assay. Representative images are shown. The area of the collagen disc was measured using Image J. The contraction degree was expressed as a percentage of the 24‐well area. The cultured HTMCs are from one human donor. Data are means ± SEM of three independent experiments. **P < .01 vs control

We then set out to determine whether AQP1 reduction was responsible for the pathological alteration of ET‐1‐induced POAG. Knock‐down of AQP1 with si‐AQP1 in HTMCs led to a collapse of actin arcs and formation of thick actin bundles (Figure 3A), alteration of intracellular and extracellular collagen (Figure 3B‐E, Figure S3), and enhancement of cell contractility (Figure 3F), which mimics the impacts of ET‐1 on HTMCs. In addition, ectopic expression of AQP1 through transfection of HTMCs with an adenoviral vector encoding for full‐length AQP1 remarkably reversed ET‐1‐induced stress fibre reorganization (Figure 3A), collagen deposition (Figure 3B‐E) and enhanced contractility (Figure 3F) in HTMCs, compared with that of vector controls. Thus, our results suggest that a decrease in AQP1 is required for ET‐1‐induced TM tissue remodelling in POAG.

### 3.4 ET‐1‐induced glaucoma tissue remodelling was mediated by the interaction between AQP1 and β‐catenin

**Figure 4:**
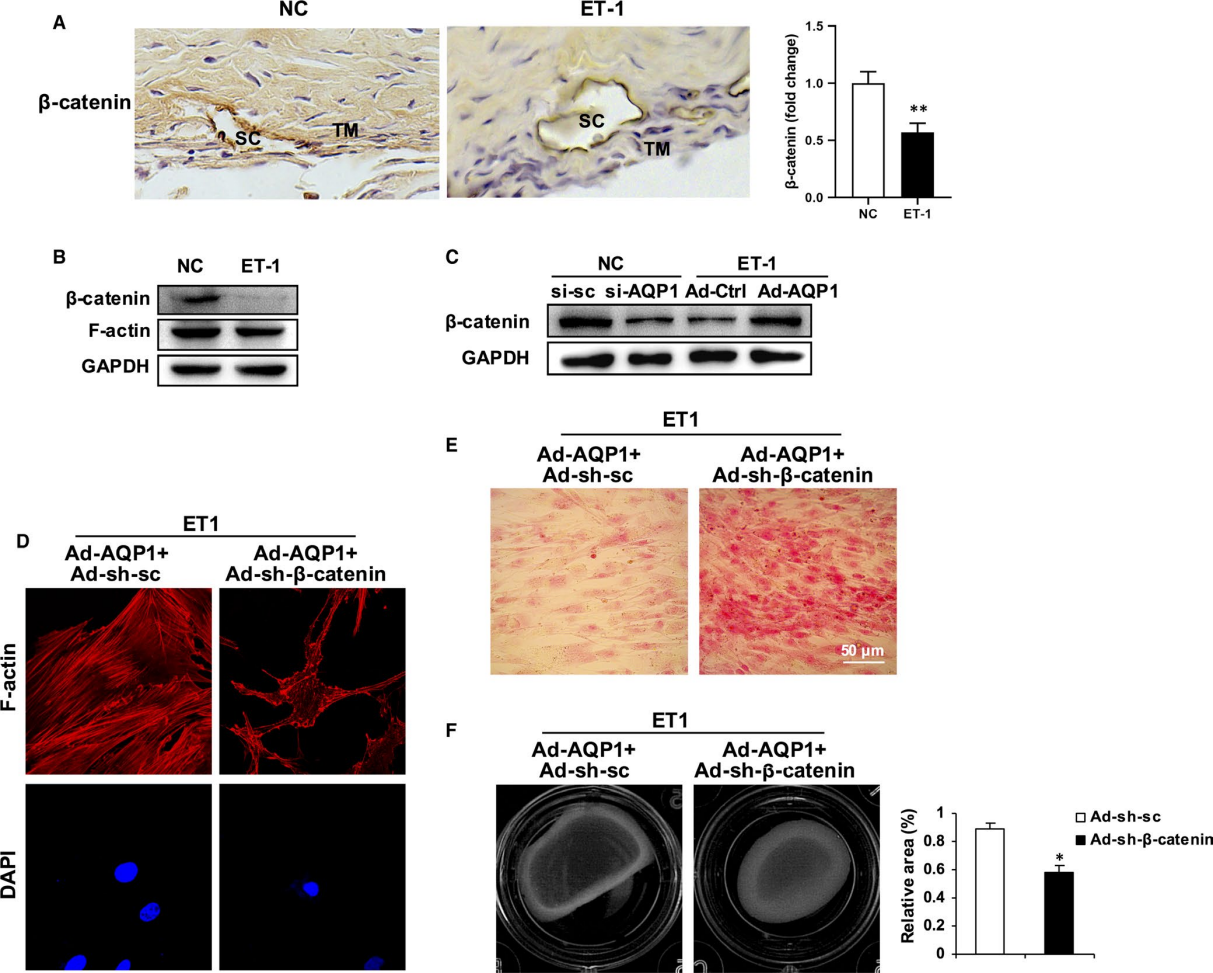
β‐catenin is required for the role of AQP1 in inhibiting ET‐1‐induced TM remodelling. A, Representative immunohistochemistry for β‐catenin in sections of rabbit trabecular meshwork tissues. TM, trabecular meshwork; SC, Schlemm's canal. B, Western blot analysis for β‐catenin and F‐actin in PBS or ET‐1 (100 nmol/L, 24 h) treated HTMCs. C, HTMCs were transfected with si‐AQP1 or scramble control si‐sc for 48 h in control HTMCs. Alternatively, cells were infected with an adenoviral vector expressing AQP1 (Ad‐Flag‐AQP1) or empty vector (Ad‐Flag). Forty‐eight hours later, cells were treated with ET‐1 for another 24 h. Protein level of β‐catenin was determined by Western blot. D‐F, HTMCs were co‐infected with an adenoviral vector expressing Ad‐AQP1 (Ad‐Flag‐AQP1) and an adenoviral vector expressing short hairpin RNA specific for β‐catenin (Ad‐sh‐β‐catenin) or scramble control (Ad‐sh‐sc). Forty‐eight hours later, cells were treated with ET‐1 for another 24 h. D, Representative immunofluorescence for F‐actin (red). Nuclei were counterstained with DAPI (blue). Scale bar, 10 μm. E, Deposition of collagen in HTMCs was determined by Sirius Red staining. F, The contractility of HTMCs was determined by a collagen gel contraction assay. Representative images are shown. The area of the collagen disc was measured using Image J. The contraction degree was expressed as a percentage of the 24‐well area. The cultured HTMCs are from one human donor. Data are means ± SEM of three independent experiments. **P < .01 vs control

AQP1 has been reported to promote cell migration through its interaction with β‐catenin.^30^, ^31^ β‐catenin is an important regulator of the Wnt signalling pathway. The interaction between AQP1 and β‐catenin mediates cytoskeletal remodelling,^32^ cell migration and proliferation,^32^, ^33^ and epithelial mesenchymal transition.^34^ Moreover, it has been found that β‐catenin signalling activation causes ECM expression and stiffness of human trabecular meshwork cells.^35^, ^36^ In order to extend our findings, we investigated the function of β‐catenin during ET‐1 exposure. Topical ocular ET‐1 treatment for 2 weeks on rabbit eyes led to a significant reduction in the level of β‐catenin (Figure 4A). Moreover, the application of ET‐1 on cultured HTMCs induced a similar β‐catenin down‐regulation effect (Figure 4B and Figure S4A). We observed that the knock‐down of AQP1 caused the β‐catenin reduction. Interestingly, AQP1 overexpression could stabilize the ET‐1 induced β‐catenin reduction (Figure 4C and Figure S4B). In order to test the role of β‐catenin in ET‐1‐induced contractility, F‐actin conformation and collagen deposition, we designed and constructed a recombinant adenovirus coding shRNA for β‐catenin (Ad‐sh‐β‐catenin) and cotransfected HTMCs with Ad‐AQP1. Compared with the ET‐1‐treated AQP1 overexpressing cells, the β‐catenin knock‐down cells were smaller and had lost elongated F‐actin arcs, as well as peripheral spreading bundles at the leading edge (Figure 4D). Moreover, images of Sirius Red staining show that collagen deposition was much more severe in the β‐catenin depleted‐HTMCs (Figure 4E). In addition, using contractility gel assay we observed that treatment with ET‐1, depletion of β‐catenin in AQP1 overexpressed HTMCs resulted in an increase of cell contractility with a reduction of the area of gel (Figure 4F). According to these results, we believe that when ET‐1 circulates in aqueous flow, a series of toxic ET‐1 effects, such as stress fibre reorganization, collagen deposition and contractility alteration, gradually affect trabecular meshwork cells. Therefore, our data show that together with β‐catenin, AQP1 plays a vital role in this process.

### 3.5 AQP1 transcription is negatively regulated by ATF4 upon ET‐1 stimulation

**Figure 5:**
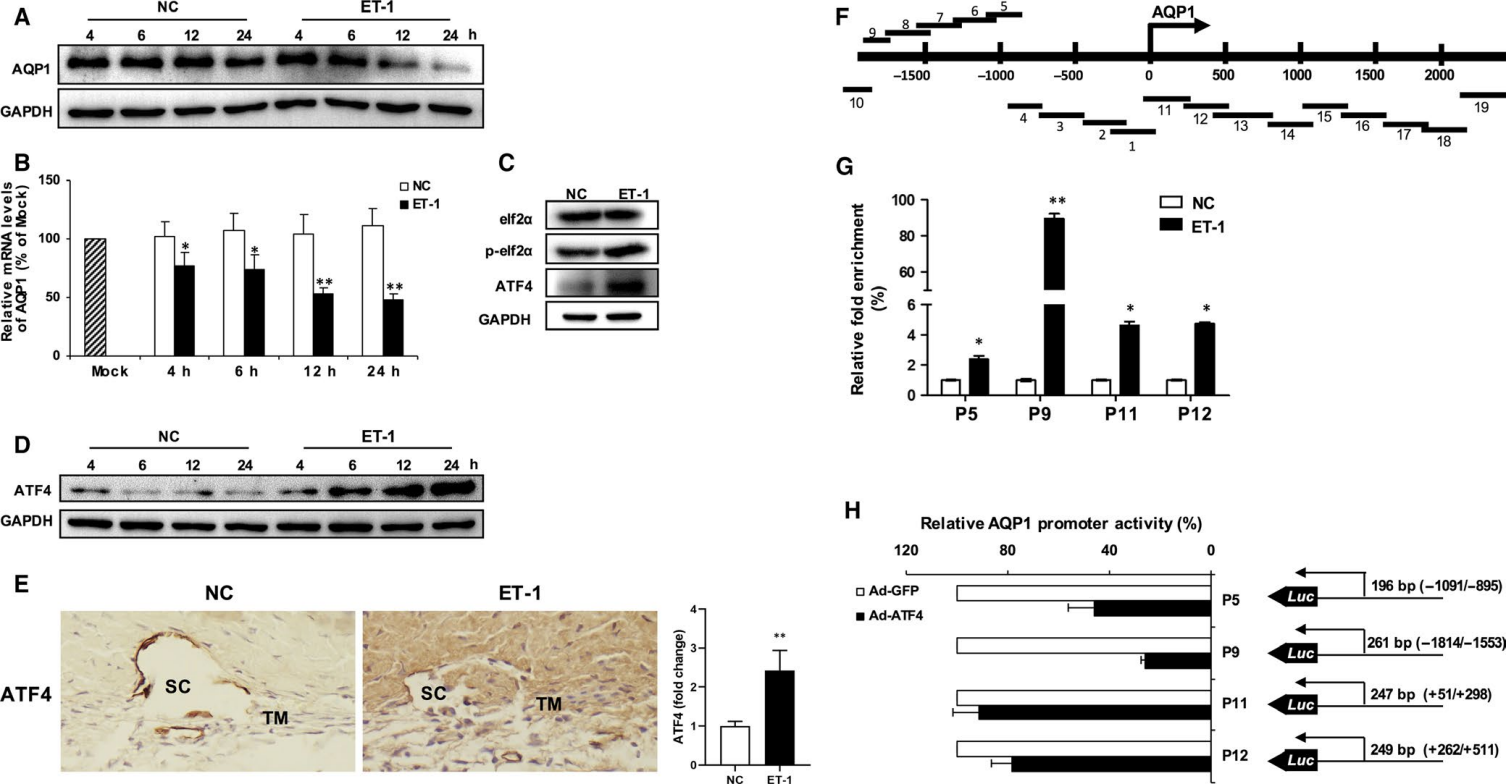
AQP1 transcription is negatively regulated by ATF4 upon ET‐1 stimulation. Protein or mRNA expression of AQP1 in HTMCs exposed to PBS or ET‐1 (100 nmol/L) for the respective 4, 6, 12 or 24 h was determined by Western blot analysis (A, the upper panel) or quantitative real‐time PCR, respectively (B, the lower panel). C, Western blot analysis for eIF2α, p‐eIF2α and ATF4 in PBS or ET‐1 (100 nmol/L, 24 h) treated HTMCs. D, Protein level of ATF4 in HTMCs exposed to PBS or ET‐1 (100 nmol/L) for the respective 4, 6, 12 or 24 h was determined by Western blot analysis. E, Representative immunohistochemistry for ATF4 in sections of rabbit trabecular meshwork tissues. TM, trabecular meshwork; SC, Schlemm's canal. F, Schematic of fragments map of AQP1 transcription start site (−2000 to +2500). G, Relative fold enrichment of ATF4 binding region of *AQP1* promoter by CHIP‐QPCR on HTMCs. Immunoprecipitated DNA was presented as percentage of total DNA input and expressed as fold changes in HTMCs treated with ET‐1 relative to PBS control. H, HTMCs were cotransfected with AQP1‐Luc reporter plasmid containing the respective AQP1 promoter region (AQP1‐P5, P9, P11 or P12) and ATF4 expression plasmids (Ad‐ATF4) for 72 h. Cells were subjected to dual‐luciferase reporter assay. Each value is shown as a percentage of the empty plasmid control (Ad‐GFP). The cultured HTMCs are from one human donor. Data are means ± SEM of three independent experiments. *P < .05 and **P < .01 vs control

We further investigated the possible mechanism underlying ET‐1‐induced AQP1 suppression. We observed that ET‐1 stimulation led to a time‐dependent inhibition of AQP1 at both protein and mRNA level (Figure 5A,B). Given that ET‐1 has been implicated in the induction of endoplasmic reticulum (ER) stress, we checked the ER stress‐related pathway using Western blotting and found that protein levels of phosphorylated eukaryotic initiation factor 2B (eIF2α) and activating transcription factor 4 (ATF4) were significantly up‐regulated by ET‐1 (Figure 5C and Figure S5A), indicating that exposure to ET‐1 activates the eIF2α/ATF4‐dependent ER stress pathway in HTMCs. In addition, ATF4 induction by ET‐1 exposure is time‐dependent (Figure 5D and Figure S5B). A significant upregulation of ATF4 was also detected in trabecular meshwork tissues from ET‐1‐treated eyes (Figure 5E). ATF4 is a potent stress response transcription factor that is a member of cyclic adenosine monophosphate responsive element‐binding (CREB) protein family.^37^ ATF4 has been found to transcriptionally up‐regulate amino acid transporter AAT (ATF4‐dependent amino acid transporter) in autophagy‐deficient tumour cells.^38^, ^39^ In order to determine whether ET‐1‐induced AQP1 suppression is transcriptionally regulated by ATF4 in HTMCs, we designed 19 primer pairs within the *AQP1* promoter region (−2000 bp to +2500 bp) (Figure 5F) and found that ATF4 can directly bind to four regions of the *AQP1* promoter (AQP1‐P5, ‐P9, ‐P11 and ‐P12) (Figure 5G). Important constructs consisting of the fragments from these four promoter regions of *AQP1* were fused to the luciferase reporter gene. Utilizing luciferase assay, we detected that the transcriptional activity of *AQP1* is negatively regulated by ATF4, suggesting that upon ET‐1 stimulation of HTMCs, ATF4 can bind directly to the *AQP1* promoter region and negatively regulate *AQP1* transcription (Figure 5H).

### 3.6 ATF4 is required for ET‐1‐induced POAG phenotypic changes in HTMCs

**Figure 6:**
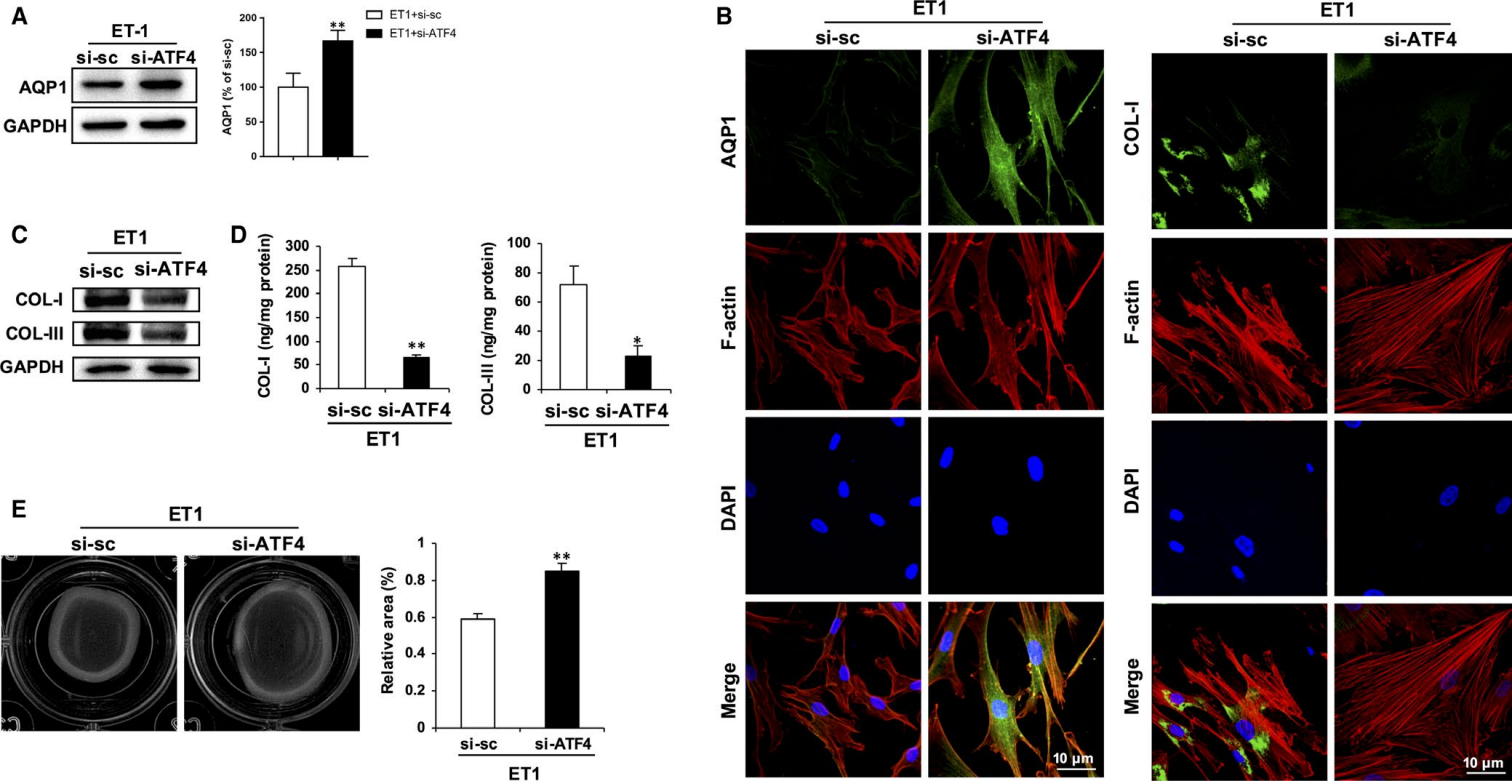
ATF4 is required for ET‐1‐induced POAG phenotypic changes in HTMCs. HTMCs were transfected with si‐ATF4 or scramble control si‐sc. Twenty‐four hours later, cells were treated with ET‐1 for another 24 h. A, Protein of AQP1 in HTMCs exposed to ET‐1 (100 nmol/L) for 24 h was determined by Western blot analysis. B, Representative immunofluorescent images depicting knock‐down of ATF4 led to increased AQP1 (green) or reduced COL‐I (green) expression, accompanied by restored F‐actin (red) arcs formation in HTMCs. Nuclei were counterstained with DAPI (blue). Scale bar, 10 μm. Deposition and secretion of collagen were determined by Western blot analysis (C) and ELISA (D), respectively. E, Collagen gel contraction assay. The cultured HTMCs are from one human donor

In order to determine whether ATF4 is a requisite of ET‐1‐triggered stiffness of HTMCs, we adopted a siRNA approach to knock‐down ATF4 (Figure 4A). Upon ET‐1 stimulation, knock‐down of ATF4 by si‐ATF4 led to the restoration of AQP1 expression (Figure 6A,B), which was accompanied by the recovery of F‐actin arcs (Figure 6B), reduction of collagen deposition and secretion (Figure 6B,C, Figure S6A,D) and cellular contractility (Figure 6E), in comparison with that of the scramble control. These data suggest that ATF4 is required for ET‐1‐induced stiffness of TM tissues in POAG, most probably through AQP1 repression.

## 4 DISCUSSION

Chronically elevated IOP is known to be the most important risk factor of POAG. Elevated IOP is implicated by disturbed AH homeostasis with increased resistance to drainage. Together with Schlemm's canal, TM facilitates the out‐flow of aqueous fluid and control of IOP in the eye. It has been observed that elevated IOP is significantly correlated with elevated ET‐1 concentrations in AH of POAG patients.^10^ However, the underlining molecular mechanism remains unknown. In our study, we made distinct progress on two aspects: (a) for the first time we observed that in the presence of ET‐1, the suppression of protein levels of water channel AQP1 causes stress fibre collapse, collagen induction, ECM remodelling and contractility alteration of the trabecular meshwork; (b) we identified ATF4 as an ET‐1 sensor in controlling TM remodelling by negatively regulating AQP1 transcription in POAG eyes.

A relatively long process of gradually elevated IOP results in patients developing POAG. TM tissue remodelling is a complicated process that includes a series of intra‐trabecular meshwork cell responses to physical and chemical stress. ET‐1, a small peptide with 21 amino acids, is synthesized and secreted by endothelial cells. In the eye, ET‐1 can be synthesized at many locations, such as the vascular endothelium, retinal pigmented epithelium and non‐pigmented ciliary epithelium. Once secreted, it can then be circulated in the anterior and posterior aqueous fluid.^6^, ^7^, ^8^ As a potent vasoconstrictor, elevated ET‐1 has been mainly implicated in many cardiovascular diseases. Nonetheless, elevated ET‐1 has also been observed in the AH of glaucoma eyes^9^ and there is significant correlation between IOP and ET‐1 concentration in AH of POAG patients.^10^, ^11^

Using rabbits as an animal model, we observed elevated IOP and AQP1 suppression in TM cells of eyes to which ET‐1 was topically applied. AQP1 is expressed in the iris and ciliary epithelium and functions in aqueous fluid production and thus IOP regulation. More importantly, AQP1 is also expressed strongly in trabecular meshwork cells and regulates the cell volume.^21^, ^22^ Our results support that AQP1 at the TM cells plays role in aqueous fluid drainage and also IOP regulation. It is worth noting that AQP1 deletion in mice reduced AH production meanwhile did not affect out‐flow of aqueous fluid.^20^ However, there are several members in the water channel family and they possess overlapped functions and can compensate with one another. In our study, the glaucomatous eye, induced by topically ET‐1 application, mimics the physiological condition better.

We noted that ET‐1‐induced AQP1 suppression is highly associated with a reduction of β‐catenin, including stress fibre reorganization, collagen deposition and contractility alteration, which leads to a series of POAG characteristic features that affect HTMCs. Primary human trabecular meshwork cells used in the study were from one human patient due to donor limitation. However, our observations in the cell culture system correlate well with the results of the animal model. β‐catenin is an important regulator of the Wnt signalling pathway. Currently available knowledge related to the effects of AQP1/β‐catenin is mainly related to cytoskeletal remodelling,^32^ cell migration and proliferation^32^, ^33^ and epithelial mesenchymal transition.^34^ Consistent with previous findings in other cell types, our study emphasizes the glaucomatous effect of AQP1/β‐catenin on the trabecular meshwork, upon ET‐1 stimulation. We showed that increased AQP1 protein levels prevent the collapse of F‐actin fibres, collagen accumulation, ECM deposition and contractility alteration of the trabecular meshwork. Furthermore, we observed that inhibition of β‐catenin signalling causes ECM expression and cell stiffness of human trabecular meshwork cells, which is consistent with results of previous studies.^35^, ^36^

ET‐1 has been reported to induce ER stress^40^, ^41^ and plays a role in inflammatory diseases, pregnancy disorders, neurodegenerative diseases and POAG.^42^, ^43^, ^44^, ^45^ Several transcription factors, such as ATF4, NFkB, eIF2a and XBP1s, are activated during ER stress.^46^ ATF4 has been shown to be induced in glaucomatous TM.^45^ ATF4 activation leads to it binding with various partner proteins and the regulation of diverse target genes. Previous study has reported AQP4 expression level was regulated by astrocytic Wnt/β‐catenin signalling in facilitating seizure induction after ischaemia^47^; however, whether the regulation was directly mediated by ATF4 was unknown. Our work implicates that activated ATF4 acts as a tissue remodelling signal upon sensing circulation of aqueous ET‐1. Upon ET‐1 treatment, activated ATF4 can bind to the promoter regions of AQP1, negatively regulate transcription of water channel AQP1 and initiate a series of steps in the glaucomatous tissue remodelling process, while the knock‐down of ATF4 protects HTMCs from ET‐1‐‐induced effects.

Our results show the harmful role of ATF4 in response to ET‐1 in HTMCs. Interestingly, ET‐1 can be synthesized and is secreted by human TM cells,^48^ and ATF4 has been reported to regulate ET‐1 transcription in human microvascular endothelia cells.^49^ It is possible that upon physical and biochemical stimulation, ET‐1 is synthesized by trabecular meshwork cells and that secreted ET‐1 binds to the receptors of neighbouring TM cells. Thus, the tissue remodelling signal is amplified. Although it seems that ATF4 turns on a positive feedback loop and leads to irreversible glaucomatous pathogenesis, we cannot exclude the possibility that it initially plays a protective role for the TM cells by protecting them from physical damage induced by elevated IOP. Therefore, further investigations are required to fully examine the physiological function of ATF4 in POAG.

**Figure 7:**
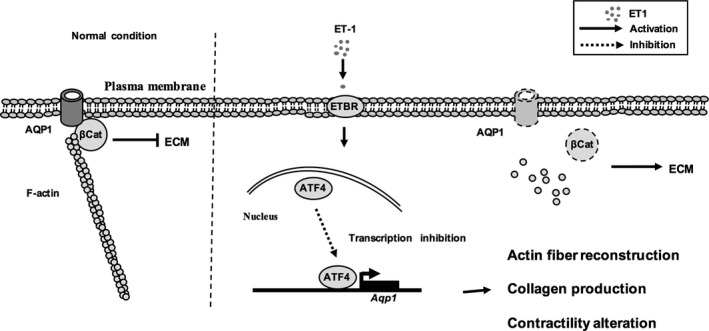
Schematic of a proposed model for tissue remodelling effects of ET‐1 on trabecular meshwork cells and the potential molecular mechanism of POAG

Taken together, our results reveal a novel role of ATF4, where it functions in the process of POAG by negatively regulating the transcription of water channel AQP1 in HTMCs (Figure 7A). This regulation is triggered by the presence of ET‐1 circulating in aqueous fluid. Additionally, for the first time we found that ET‐1‐induced suppression of AQP1 contributes to alteration of trabecular meshwork tissue, including intracellular cytoskeleton collapse, collagen production, extracellular collagen deposition and contractility alteration of trabecular meshwork cells. Thus, our study provides a brand new direction for developing potential pharmaceutical targets for the prevention or treatment of POAG.

## CONFLICT OF INTEREST

No potential conflicts of interest were disclosed.

## AUTHOR CONTRIBUTIONS

The authors contributed in the following way: YZ and YY conceived the study and designed the experiments. HZ performed the experiments with the help of YZ, XH, XS, XS, GL, XC, YY, JM, LC and XW. YZ, HZ and YY interpreted data and contributed to discussion. YZ and YY drafted the manuscript.

## Data Availability

All data generated or analysed during this study are included in this article.
