# Supplementary figures and images for: AQP1 suppression by ATF4 triggers trabecular meshwork tissue remodelling in ET‐1‐induced POAG
Source: J Cell Mol Med. 2020 Feb 13;24(6):3469–80. doi: 10.1111/jcmm.15032 (PMC7131939; doi:10.1111/jcmm.15032)

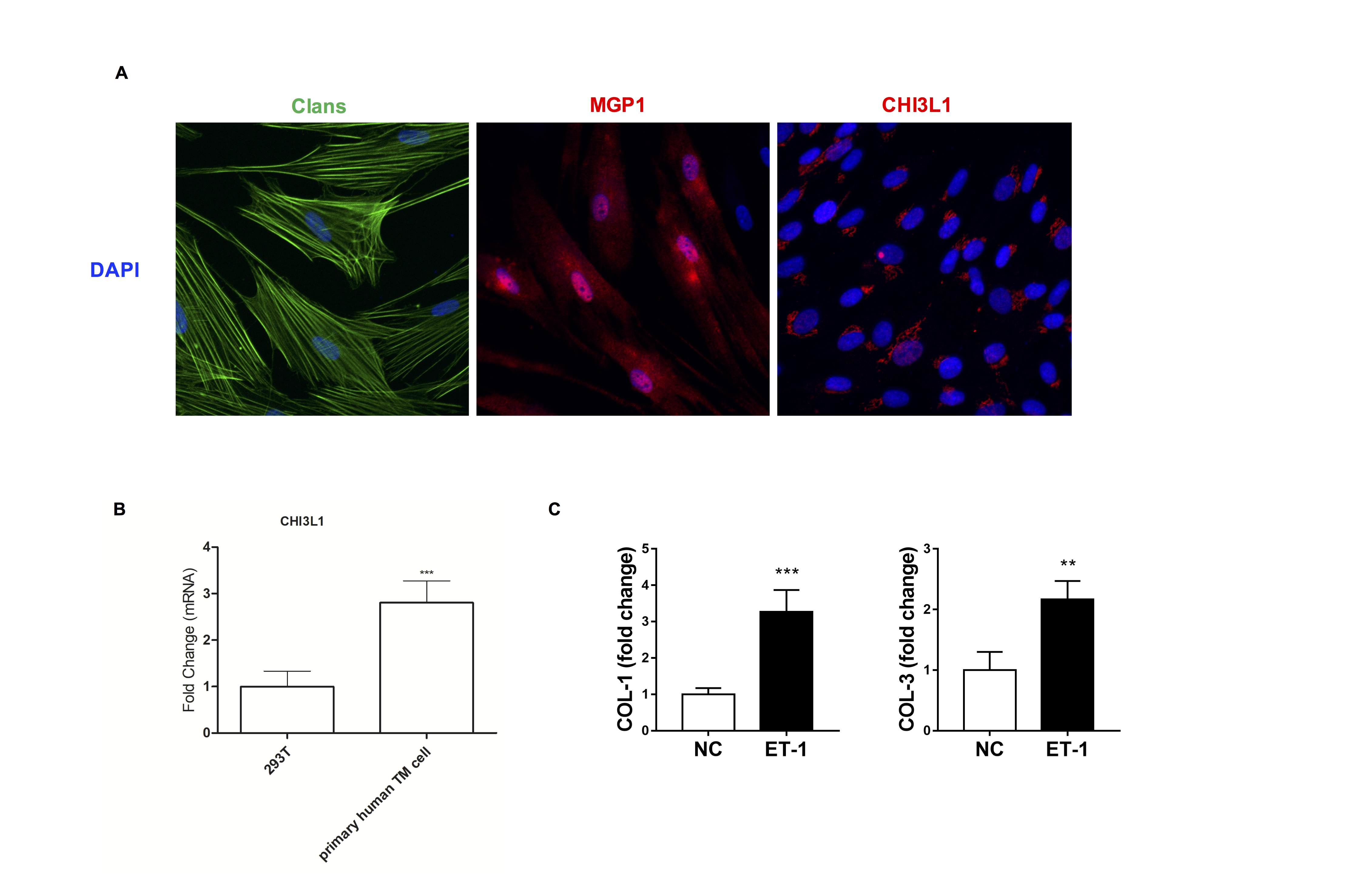

Supplement: Supplementary file 1 [file JCMM-24-3469-s001.tiff]

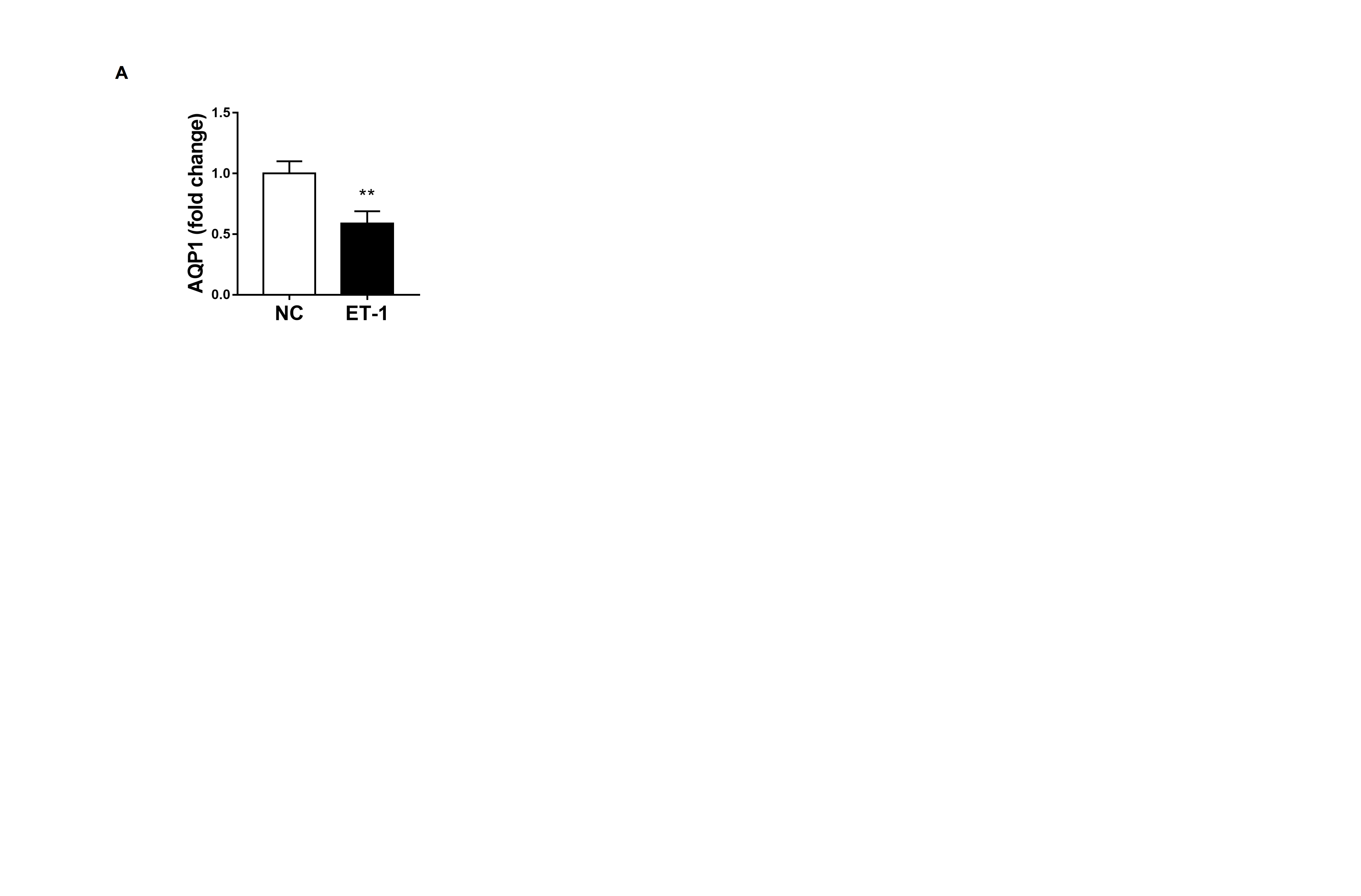

Supplement: Supplementary file 2 [file JCMM-24-3469-s002.tiff]

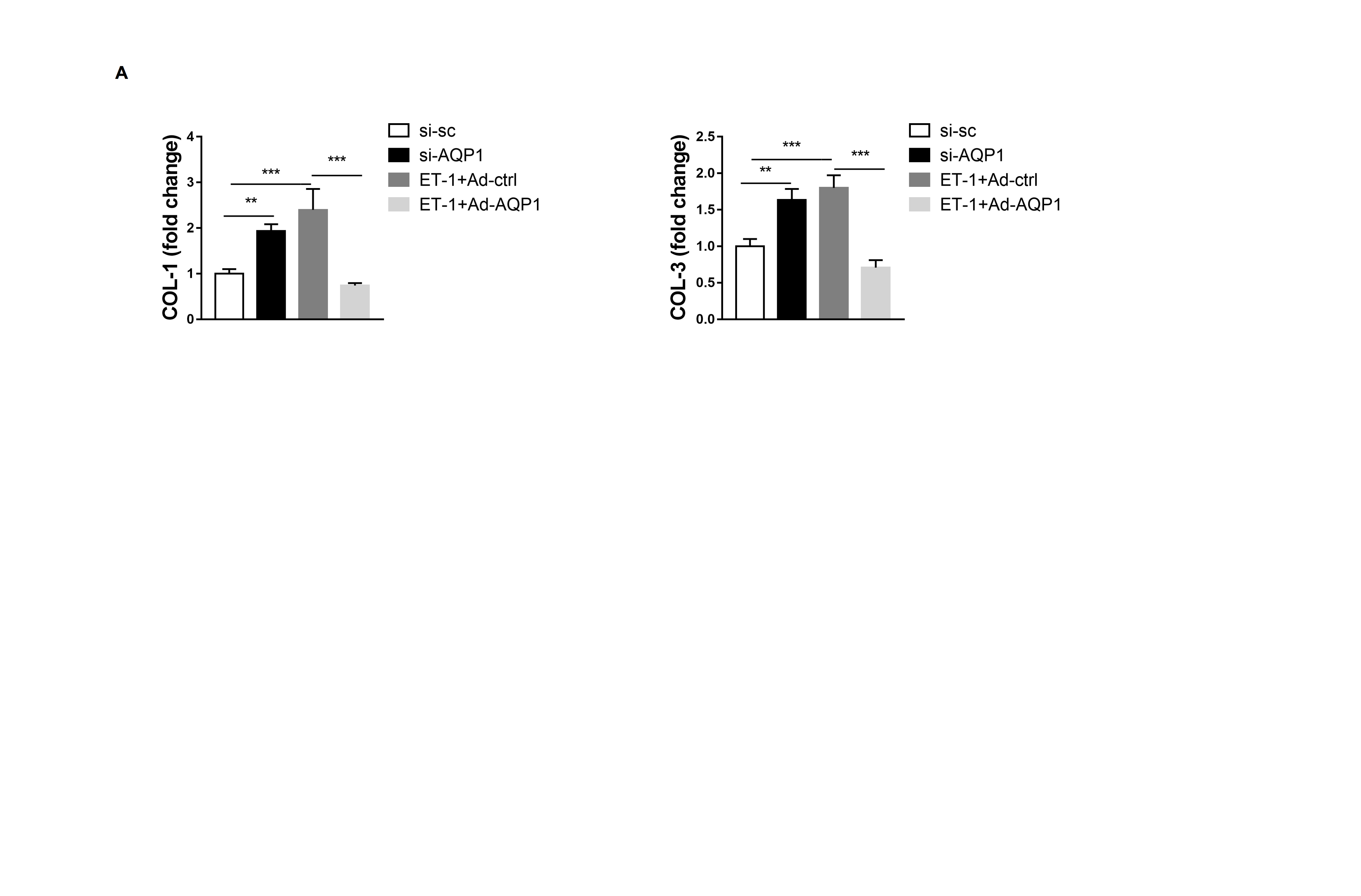

Supplement: Supplementary file 3 [file JCMM-24-3469-s003.tiff]

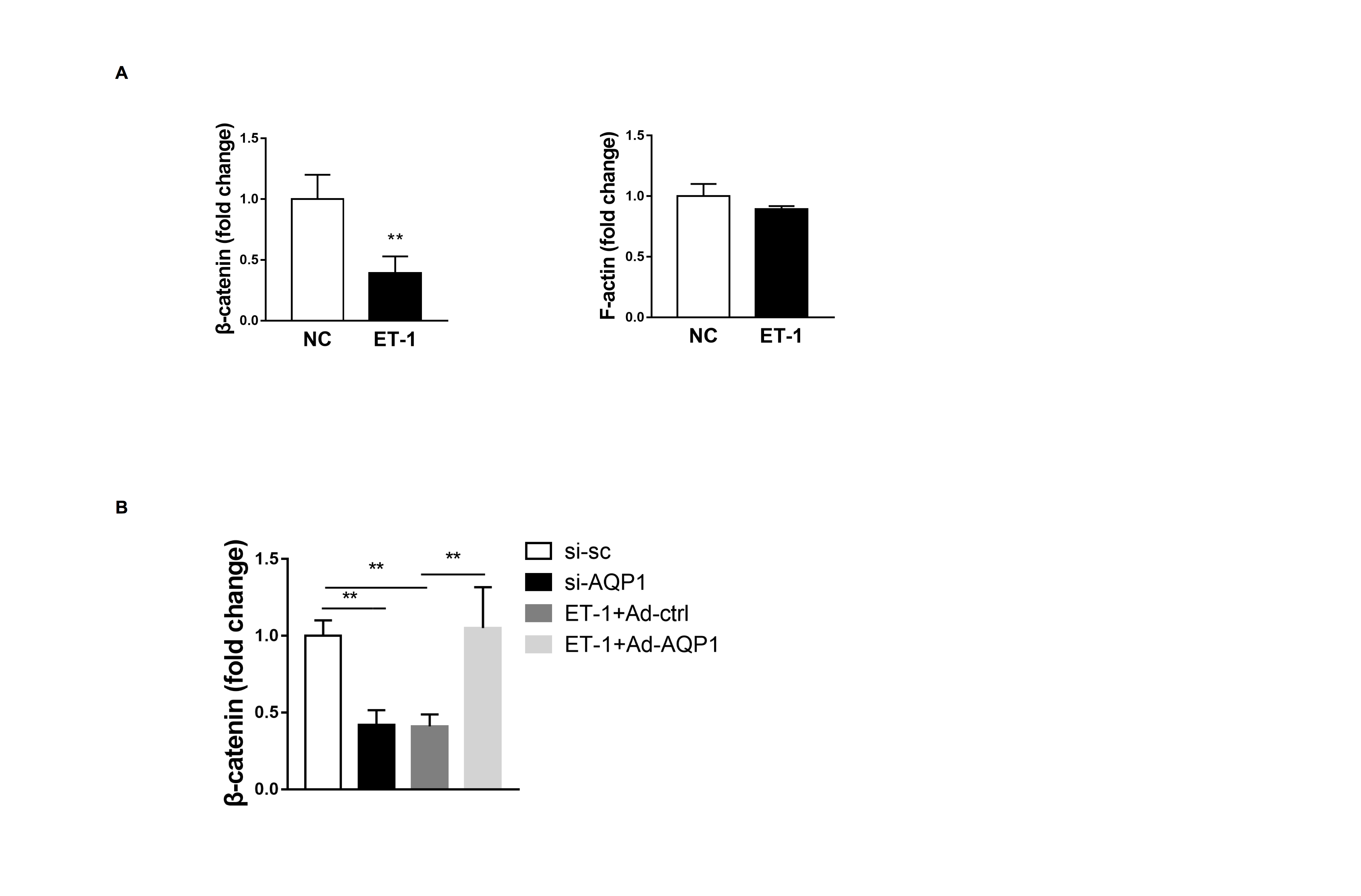

Supplement: Supplementary file 4 [file JCMM-24-3469-s004.tiff]

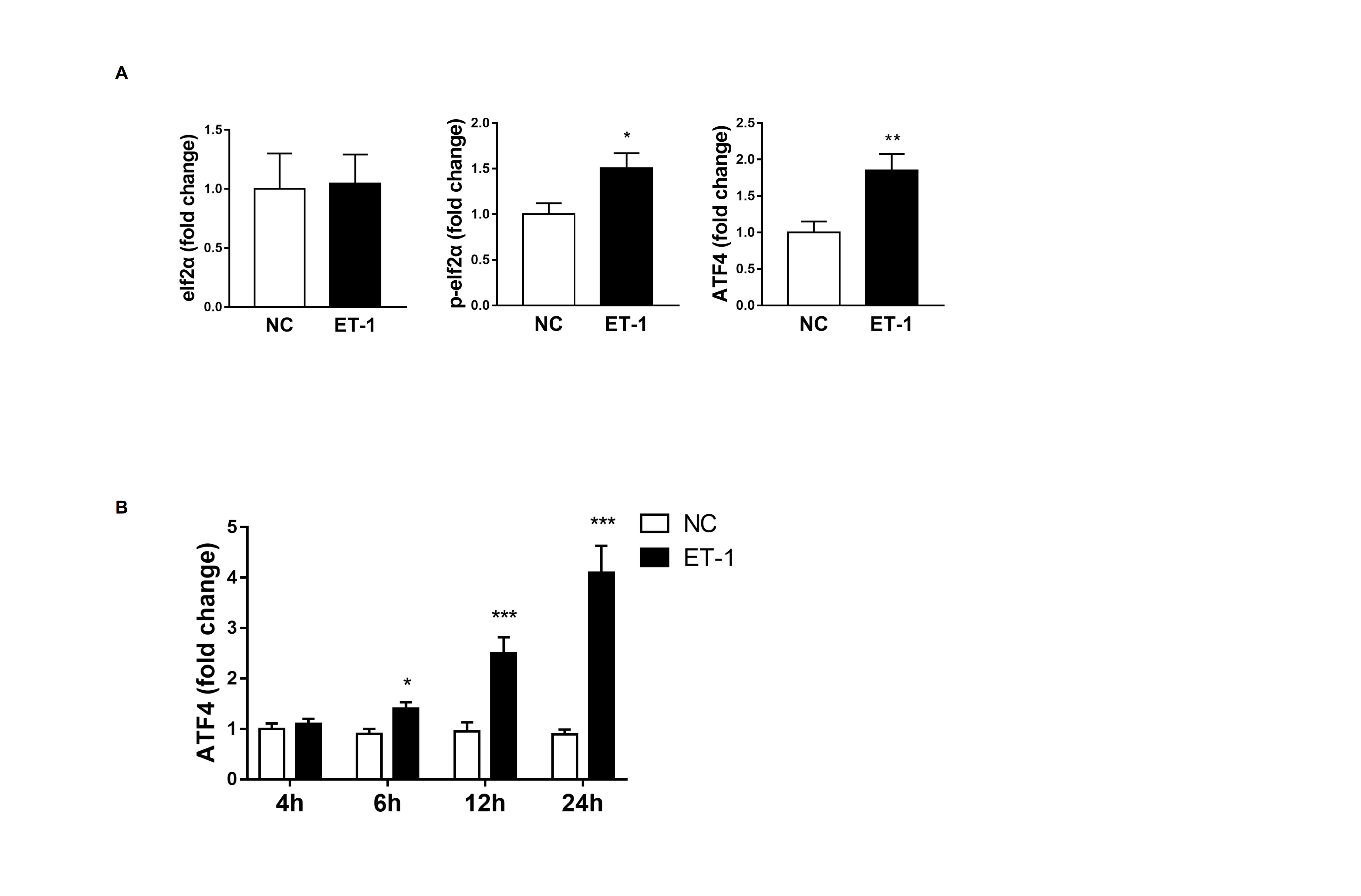

Supplement: Supplementary file 5 [file JCMM-24-3469-s005.tiff]

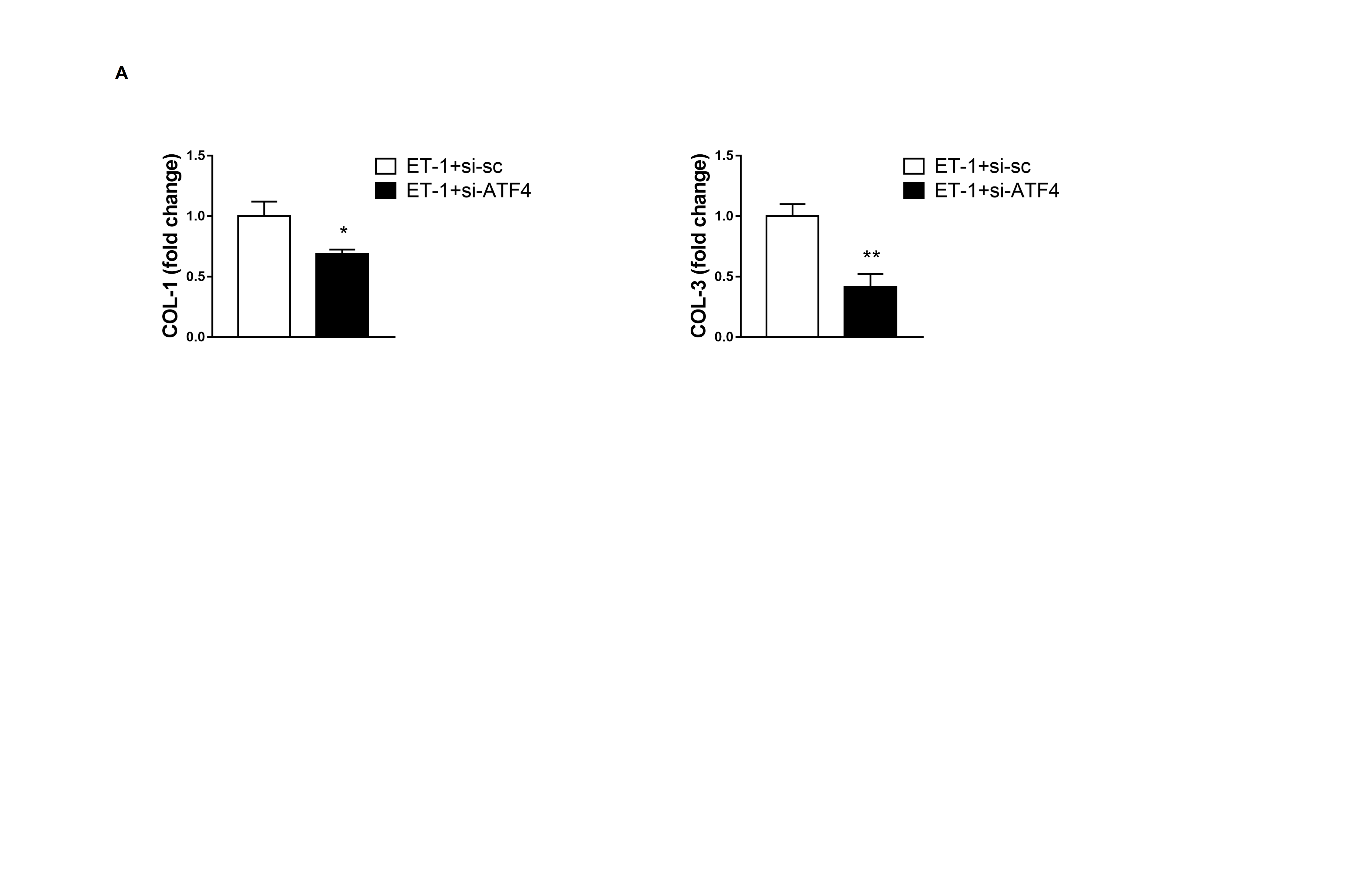

Supplement: Supplementary file 6 [file JCMM-24-3469-s006.tiff]
