# Supplementary material for: AQP1 suppression by ATF4 triggers trabecular meshwork tissue remodelling in ET‐1‐induced POAG
Source: J Cell Mol Med. 2020 Feb 13;24(6):3469–80. doi: 10.1111/jcmm.15032 (PMC7131939; doi:10.1111/jcmm.15032)
Supplement: Supplementary file 7 [file JCMM-24-3469-s007.docx]

Supplemental Figure 1

(A). The primary human TM cell line were confirmed with immunofluorescent staining with three specific markers: Clans, MGP1 and CHI3L1. (B). mRNA level of CHI3L1 in the primary TM cell line was tested by RT-PCR comparing with 293T cell line. ^***^*P* < 0.001 vs Mrna of 293T cell line. (C). Deposition collagen in cultured HTMCs were determined by Western Blot. Western Blot results were quantified using Image J. Data are means ± SEM of three independent experiments. ^**^*P* < 0.01 and ^***^*P* < 0.001 vs PBS control (NC).

Supplemental Figure 2

(A). Protein of AQP1 in HTMCs exposed to PBS or ET-1 (100 nM) for 24 hours was determined by Western blot analysis. Western Blot results were quantified using Image J. Data are means ± SEM of three independent experiments. ^**^*P* < 0.01 vs PBS control (NC).

Supplemental Figure 3

(A). Protein expression of collagen I (COL-I) and III (COL-III) was determined by Western blot. Western Blot results were quantified using Image J. Data are means ± SEM of three independent experiments. ^**^*P* < 0.01 and ^***^*P* < 0.001vs si-sc; ^***^*P* < 0.001 vs Ad ctrl.

Supplemental Figure 4

(A). Western blot analysis for β-catenin and F-actin in PBS or ET-1 (100 nM, 24 h) treated HTMCs. Western Blot results were quantified using Image J. Data are means ± SEM of three independent experiments. ^**^*P* < 0.01 vs PBS control (NC). (B). Western blot analysis for β-catenin Protein level of primary TM cells infected with si-AQP1, scramble control si-sc, adenoviral vector expressing AQP1 (Ad-Flag-AQP1) or empty vector (Ad-Flag). Western Blot results were quantified using Image J. Data are means ± SEM of three independent experiments. ^**^*P* < 0.01 vs si-sc; ^**^*P* < 0.01 vs Ad ctrl.

Supplemental Figure 5

(A). Protein level of eIf2a, p-eIf2a and ATF4in HTMCs exposed to PBS or ET-1 (100 nM) was determined by Western blot analysis. Western Blot results were quantified using Image J. Data are means ± SEM of three independent experiments. ^*^*P* < 0.05 vs PBS control (NC). (B). Protein level of ATF4in HTMCs exposed to PBS or ET-1 (100 nM) for the respective 4, 6, 12, or 24 hours was determined by Western blot analysis. Western Blot results were quantified using Image J. Data are means ± SEM of three independent experiments. ^*^*P* < 0.05 and ^***^*P* < 0.001 vs PBS control (NC).

Supplemental Figure 6

(A). Protein of Col-I, COL-III in HTMCs, transfected with si-ATF4 or scramble control si-sc with 24 hours ET-1 (100 nM) exposure, determined by Western blot analysis. Western Blot results were quantified using Image J. Data are means ± SEM of three independent experiments. ^*^*P* < 0.05 and ^***^*P* < 0.001 vs ET-1+si-sc
